# The effect of mindfulness-based stress reduction on rejection sensitivity and resilience in patients with thalassemia: a randomized controlled trial

**DOI:** 10.1186/s12888-023-04802-z

**Published:** 2023-04-21

**Authors:** Asma Ghonchehpour, Mansooreh Azizzadeh Forouzi, Mahlagha Dehghan, Atefeh Ahmadi, Gloria Okou, Batool Tirgari

**Affiliations:** 1 Department of Medical Surgical Nursing, Razi Faculty of Nursing & Midwifery, Kerman University of Medical Sciences, Kerman, Iran; 2 Neuroscience Research Center, Institute of Neuropharmacology, Kerman University of Medical Science, Kerman, Iran; 3 Department of Critical Care Nursing, Razi Faculty of Nursing and Midwifery, Kerman University of Medical Sciences, Kerman, Iran; 4 Department of Counselling in Midwifery, Razi Faculty of Nursing and Midwifery, Kerman University of Medical Sciences, Kerman, Iran; 5 Department of Nursing, Mount Saint Mary University, Los Angeles, CA United States; 6 Nursing Research Center, Kerman University of Medical Sciences, Kerman, Iran

**Keywords:** Mindfulness-based stress reduction, Rejection sensitivity, Resilience, Thalassemia

## Abstract

**Background:**

Thalassemia is a genetic and chronic congenital disorder composed of physical problems that severely impair patients’ cognitive, psychological and social processes. The rehabilitation of patients is particularly important because they have a high rejection sensitivity and low resilience. The present study aimed to determine the effects of mindfulness-based stress reduction counseling on rejection sensitivity and resilience in patients with thalassemia referring to a dedicated disease center in Kerman, Iran.

**Materials and methods:**

We conducted this randomized controlled trial study on 66 patients with thalassemia referring to the Kerman Thalassemia Center in Kerman, Iran in 2022. Using convenience sampling and the stratified block randomization method, we divided the samples into two intervention (*N* = 33) and control (*N* = 33) groups. Patients in the intervention group received eight 60-min online mindfulness-based stress reduction counseling sessions (one session per week) and completed the Rejection Sensitivity Questionnaire, Adult Version (A-RSQ) and the Conner-Davidson Resilience Scale before and after the intervention. We collected data using the SPSS 25 trial and descriptive statistics (frequency, percentage, mean and standard deviation), Chi-Square test, Independent-samples t-test, Fisher's exact test, and Analysis of covariance. A significance level of 0.05 was considered.

**Results:**

We found no significant difference in the mean scores of rejection sensitivity between the intervention (8.75 ± 4.86) and control groups (9.87 ± 5.16) before the intervention. Mean scores for rejection sensitivity were 10.23 ± 4.94 in the control group and 7.11 ± 4.13 in the intervention group after the intervention, the results of analysis of covariance showed that, there was a significant difference between two groups after the intervention (F = 7.52, *p* = 0.008). The mean resilience score in the control group was 63.69 ± 19.43, while it was 67.72 ± 17.98 in the intervention group before the intervention and there is no significant difference between them, but the mean resilience scores in the control and intervention groups were 58.06 ± 22.81 and 74.18 ± 17.46 after the intervention, respectively. the results of analysis of covariance showed that, there was a significant difference between two groups after the intervention (F = 9.28, *p* = 0.004).

**Conclusion:**

Our results showed that in addition to other physical treatments, mindfulness-based stress reduction counseling was effective in reducing the patient’s rejection sensitivity and increasing the resilience of patients with thalassemia.

## Introduction

Thalassemia is a genetic and chronic congenital disorder in the world that causes mild or major anemia [1–4]. Three percent of the world's population (150 million people) live with thalassemia, and in 2019, there were 30,000 people living with thalassemia in Iran, with the number increasing by about 1,000 people each year [5], so Iran ranks first in the world in terms of the ratio of people with thalassemia to the total population [6]. Thalassemia is a heterogeneous grouping of genetic disorders [7] caused by decreased hemoglobin production (δβγ and βδ, β, α) and is classified into two types: alpha thalassemia and beta thalassemia. Beta thalassemia is a group of hereditary blood disorders characterized by anomalies in the synthesis of beta chains of hemoglobin and has three main forms- minor, intermedia and major [8]. Despite significant advances in medicine, thalassemia major still carries a high risk of mortality, and if the patient receives no regular transfusion, his/her survival rate will be very low [2, 4]. Prolonged and frequent treatment regimens and visits of these patients for transfusion and their chronic pains affect different aspects of their lives [4, 9]. The patients may experience headache, weakness, premature fatigue, hair loss, severe musculoskeletal pain, and short stature [4, 10]. Thalassemia and its treatment are associated with many psychological problems: anxiety, depression, isolation, and emotional disorders [11]. Studies have suggested that 80% of the patients with thalassemia have at least one psychological disorder, such as depression, anxiety, psychosomatic illness, social problems and isolation. Emotional disorders can increase rejection sensitivity and decrease resilience in these patients [3, 4, 12].

Rejection sensitivity refers to anxious expectations of rejection in situations where there is a possibility of rejection by close people [13]. A person with a high rejection sensitivity overreacts, as if an allergic reaction were occurring, so his/her defense mechanism quickly becomes alert and he/she shows aggression and hostility [12, 14]. These individuals interpret vague and unclear signals from others as rejection signs and respond aggressively [14].

Reduced resilience is another problem experienced by patients with thalassemia [3, 15]. Resilience refers to maintaining proper functioning, avoiding underlying stressors, and successfully adapting to difficult life experiences. Therefore, it is useful not only to be secure in the face of challenges [10, 16], but also to develop learning and growth in difficult living conditions and to determine how people respond to change. [17]. Resilience improves social adaptation and mental health of people in different situations. Resilient people are able to strengthen feelings of empathy and communicate better with others, improve their functional capacity and social communication and manage their lives [3]. Yoosefian et al. (2020) [3]  found that the resilience of patients with thalassemia was below average [3].

Healthcare providers have focused on the physical treatment of thalassemia patients, neglecting their psychological treatments [3, 6, 11]. Considering the impact of various psychological factors on patients with thalassemia, it is necessary to use non-pharmacological methods to improve the psychological state of these people [9]. Psychological interventions lead to the acquisition or improvement of effective coping strategies to reduce symptoms and emotional problems [6]; Mindfulness-Based Stress Reduction (MBSR) is a program that has proven useful for psychological symptoms over the past two decades [4, 9].

Kabat-Zinn founded the mindfulness-based stress reduction clinic at the University of Massachusetts Medical Center [18]. By creating self-awareness and strengthening the components of attitude, intention, and attention, mindfulness training enables a person to control his/her judgmental, objective state, as well as his/her feelings and emotions [4] A mindful person tries to gain insight into others’ thoughts, emotions and interactions and choose useful responses purposefully [9]. Mindfulness counseling trains people to become aware of their breathing (focus on the breath and observe thoughts without getting caught up in them), to scan their body (increased awareness and acceptance of sensations in different body parts), to understand their painful and negative feelings without suppressing them or preventing people from living a meaningful life and achieving their goals [19]. Kabat-Zinn lists 9 components of mindfulness that are designed for MBSR such as non-Jugging, compassion, patience, not struggling, acceptance, trust, beginner mind, letting go and, kindness [20]. Mindfulness components such as non-Jugging, acceptance and self-compassion can lead to non-attachment, which itself plays a role in reducing rejection sensitivity. Also, programs based on mindfulness are based on collective awareness, which itself is an opportunity to rebuild a sense of belonging and participation, which can lead to a connection between mindfulness and rejection sensitivity and its reduction [21]. Also, Mindfulness changes how people look at life and improves the quality of communication with themselves, the world around them, and others with compassionate and realistic acceptance. Therefore, mindfulness can reduce negative psychological symptoms and increase resilience [22]. In mindfulness, instead of avoiding them, the person is encouraged to face and accept them, and it causes people's resilience to increase [23].

Some studies addressed the effect of mindfulness-based stress reduction on resilience, life expectancy [24], quality of life [25], pain self-efficacy [9], pain and psychological symptoms [4], self-discrepancy [26], and post-traumatic stress disorder [27]. Akbari et al. (2022) [25] reported the effect of the mindfulness-based stress reduction program on the quality of life in patients with thalassemia [25]. Raisi et al. (2020) [9] indicated the effect of mindfulness-based stress reduction training on pain self-efficacy in patients with thalassemia major and suggested it in addition to drug therapy [9]. Naghibi et al. (2020) [4] found that mindfulness-based stress reduction had a significant effect on the reduction of anxiety, depression and pain intensity in patients with thalassemia (4). Jabbarifard et al. (2019) [15] indicated that mindfulness-based cognitive therapy had a significant effect on perceived stress, resilience and quality of life of patients with thalassemia major [15]. Adelian et al. (2021) [18] demonstrated the impact of mindfulness-based stress reduction on the resilience of vulnerable women referring to drop-in centers in southeastern Iran [18]. According to the study by Joss et al. (2020) [21]—nonattachment predicts empathy, rejection sensitivity, and symptom reduction after mindfulness-based intervention among young adults with a history of childhood maltreatment- mindfulness-based intervention reduces the rejection sensitivity score, posttraumatic stress disorder, and increase non-attachment and mindfulness [21].

Patients with thalassemia suffer from many problems, such as rejection sensitivity, which can be influenced by different cultures [14], and low resilience, which can affect all aspects of the life and cause many physical and mental health problems for patients with thalassemia and their families. We found limited studies on the effect of mindfulness-based stress reduction on rejection sensitivity and resilience in Iran and the world, so we decided to investigate the impact of mindfulness-based stress reduction counseling on rejection sensitivity and resilience of the patients with thalassemia referring to the dedicated disease center in Kerman, Iran in 2022.

### Hypothesis

Mindfulness-based stress reduction has an effect on rejection sensitivity and resilience of patients with thalassemia**.**

## Methods

### Study type and setting

This randomized controlled trial study was conducted on patients with thalassemia referring to the Kerman Thalassemia Center in Kerman, Iran in 2022. The study setting was the Kerman Thalassemia Center that provided transfusion and health care services for patients with thalassemia from different parts of Kerman province. Kerman is located in the southeast of Iran.

## Sample size and sampling

Inclusion criteria were patients with thalassemia major [4] aged 18 to 65, who had access to an Android smartphone, computer or the internet to participate in a virtual counseling, with at least elementary education [9], no psychologically diagnosed disorders under treatment [3, 4], and no other psychological intervention received [4]. Exclusion criteria were patients who were absent from more than two sessions [4], incompletely filled in questionnaires (more than 10% of the total questions in the questionnaire) [9], did not perform or submit any practices via WhatsApp [3], were from a neighboring province and had an acute and newly diagnosed condition [6] (according to the medical record).

**Fig. 1:**
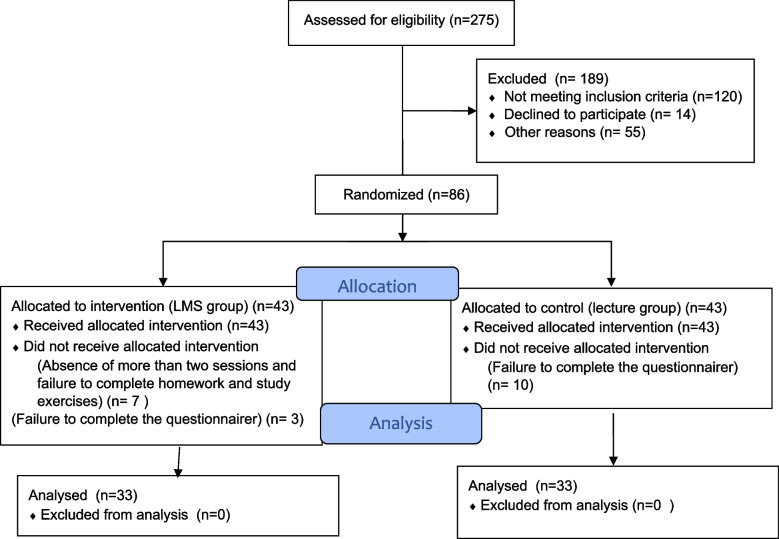
Study flow diagram: recruitment and allocation to study groups

As the study population was limited all patients with thalassemia were assessed for eligibility criteria. Then the eligible samples were allocated into 2 groups by stratified block randomization method (stratum: gender and age ± 2). Labels A and B (A = intervention and B = control) were assigned to the groups, and the block size was 4. The randomization list was generated using free online software (https://www.sealedenvelope.com/simple-randomiser/v1/lists). The third author generated the randomization list, and the first author enrolled the participants and assigned them to 2 groups. The researcher examined 275 patients with thalassemia major aged above 18 years in terms of inclusion criteria, but only 100 patients were eligible to be included in the study. Fourteen patients were excluded due to unwillingness to participate in the study, so 86 patients participated in the study and were assigned into two intervention and control groups (43 patients in each group). We excluded 10 samples from the control group due to incomplete questionnaires, and 10 samples from the intervention group due to unfavorable physical conditions, absence from more than two sessions, failure to do practices or complete the post-test questionnaire. Power analysis calculations with G*Power software version 3.1.9.2. indicated that (power = 80%, *P* = 0.05, number of groups = 2, number of measurements = 2, and number of covariates = 1) 64 participants would be needed to detect an effect size of 0.4. Finally, 33 patients in the intervention group and 33 patients in the control group were analyzed (Fig. 1).

## Data collection tools

We used demographic and background characteristics form, the Rejection Sensitivity Questionnaire, Adult version (A-RSQ) and the Conner-Davidson resilience scale in this study.

## Demographic and background characteristics

The personal characteristics of patients included age, gender, marital status, education level, occupation, number of children. The background characteristics information included the average length of stay in the hospital in the last year, the age of the first transfusion, the number of transfusions in the last year, and history of chronic diseases.

## Rejection Sensitivity Questionnaire, Adult version (A-RSQ)

Downey and Feldman [28] developed this questionnaire to measure rejection sensitivity [28], and it was standardized in Iran [29]. This questionnaire includes 9 two-part situations (A and B) in which people ask things of others. For each item, imagine that you are in the situation and then answer the questions on a six-point Likert scale from one (very unconcerned) to 6 (very concerned) in part A and from one (very unlikely) to six (very likely) in part B. The answers of the participants to the hypothetical situations are based on two dimensions: degree of anxiety and concern about the outcome and expectations of acceptance or rejection. Part A- degree of anxiety and concern—is about the level of anxiety that a person feels in the situation related to that question, while part B- expectancy of acceptance- evaluates the probability of receiving a positive answer from others. The questions in part A directly measure the respondent's anxiety and concern about rejection, but the questions in part B only measure the possibility and expectation of rejection rather than a person's concern and anxiety. The degree of rejection sensitivity is calculated by first subtracting the scores in part B from the number 7, and then multiplying the expected likelihood of rejection for each situation by the degree of anxiety, so the mean score for 9 situations is obtained. The total score of rejection sensitivity will be the mean score of rejection sensitivity in 9 situations, ranging from 1 to 36 [30], with higher scores indicating higher rejection sensitivity [29]. Berenson et al. [31] reported the Cronbach's alpha coefficient of 0.89 and the test–retest reliability of 0.91 for this questionnaire, indicating the good reliability and validity of this questionnaire [31]. Karamlou et al. (2016) [32] psychometrically measured this questionnaire in Iran, confirmed its validity and reported Cronbach's alpha coefficient of 0.81 [32]. In the present study, the Cronbach’s alpha coefficient was obtained 0.73.

## Conner-davidson resilience scale

The Conner-Davidson resilience scale (2003) was used to measure resilience [33], which was standardized in Iran as well [34]. This 25-item scale is rated on a four-point Likert scale from zero (completely false) to four (always true); the scores range from 0 to 100, with higher scores indicating greater resilience. Conner-Davidson reported the Cronbach's alpha coefficient of 0.89 for American participants [35]. Mohammadi et al. (2006) [34] standardized this scale in Iran, confirmed its validity as well as its reliability (0.89) using Cronbach's alpha coefficient [34]. In the present study, the Cronbach’s alpha coefficient was obtained 0.92.

## Data collection

After obtaining the necessary permits, the researcher visited the Kerman thalassemia center and started sampling. The present study lasted from April to mid-August 2022. She visited the thalassemia center in the morning and invited all eligible patients with thalassemia to participate in the study. She explained them the study objectives and method and received their informed consent. The participants were divided into two control and intervention groups using a stratified block randomization method (43 in each group). The demographic characteristics information form, the rejection sensitivity questionnaire-adult version, and the Conner-Davidson resilience scale were completed by both groups before the intervention (online questionnaire https://porsline.ir/ online-questionnaire). The researcher used WhatsApp to explain participants how to complete an online questionnaire; the spread of COVID-19 was the reason of using an online questionnaire. She created a WhatsApp group for the intervention group and explained them how to do the intervention, enter the sessions, and do the practices. The intervention group received eight 60-min online counselling sessions [9] once a week [4] through the Adobe Connect link. Table 1 illustrated the content of this training during 8 sessions. The first author was under the supervision of a faculty member specialized in counseling (the fourth author) for three months and could receive certificate regarding this type of intervention. The first author answered participants’ questions regarding the practices and posted offline videos on the WhatsApp. The research team reviewed the participants’ assignments one week after the end of the sessions and gave them feedback individually. The participants of the intervention group were asked not to exchange information until the end of the study. The intervention group completed the rejection sensitivity questionnaire and the Conner-Davidson resilience scale after the intervention, which were sent to the patients’ WhatsApp numbers through the link made in the Porsline website. The control group received only routine care and could access offline counseling sessions after the intervention.

**Table 1:** Mindfulness-based stress reduction counseling sessions

Sessions	The content of mindfulness-based stress reduction counseling sessions
1	Introductory session, goal setting, definition of key research variables, awareness of the meaning and concept of mindfulness, nonjudgmental observation, fluid flow of external and internal stimuli, freedom from automatic pilot, raisin meditation Assignment: eating meditation (concentration of what we do at home)
2	Discussing homework, reviewing the difference between current and previous sessions, discussing obstacles to practice (restlessness and mind-wandering), exploring our edges, and coming home to our bodies, knowing the body and its emotions and stress reactions, taking more care of ourselves (fight or flight) Body scan practice (body meditation), sitting meditation and breathing Assignment: All previous assignments plus new assignment (sitting meditation)
3	Discussing the previous assignment, focusing on being in the present (coming home to our bodies), observing and hearing without judgement (2 min), and focusing on the five senses (3 min) Assignment: sitting meditation, body scan, breathing, conscious observation and listening in an unpleasant event and alternative behaviors
4	Discussing previous practices, defining stress and the body's reaction to it, paying attention to the body sounds, breathing, breathing and thoughts, responding and reacting to difficult situations and walking consciously Assignment: previous practices, a three-minute practice in an unpleasant event and alternative behaviors of walking consciously
5	Discussing the previous practices, starting the second phase of mindful body movements and responding more effectively to stress, mediating in daily life, becoming aware of thoughts and feelings and saving more energy to deal with problems Assignment: previous practices, a three-minute practice in an unpleasant event
6	Discussing previous practices, doing homework in groups of 3 and practicing successive thoughts techniques for an hour, confronting others, and Mindful communication. Content of thoughts are not real, and what is the best way to take care of ourselves? Assignment: doing a combination of practices, a three-minute practice in an unpleasant event and a new activity
7	Discussing previous practices and taking more care of ourselves (life is like a game of snakes and ladders, rebalance in life) Four-dimensional meditation, pleasant and unpleasant events and how to make events pleasant through a three-minute mindful practice Assignment: combined techniques, a three-minute practice of an unpleasant event and a new technique
8	Doing previous practices in nature (mountain meditation), discussing about goals, character development and coping skills

## Data analysis

The SPSS25 trial version was used for data analysis; descriptive statistics (frequency, percentage, mean and standard deviation) were used to describe the demographic and background characteristics of the participants. Chi-square test, independent samples t-test and Fisher’s exact test were used to determine the homogeneity of two groups in terms of demographic variables. Shapiro–Wilk test was used to determine the normality of the resilience and rejection sensitivity variables (the sum of the two questionnaires). In addition, Levene’s test of equality of error variances fulfilled for both scores of rejection sensitivity and resilience after the intervention. Analysis of covariance was used to determine the difference between two groups regarding rejection sensitivity and resilience scores after the intervention by controlling the pretest scores. The significance level in this study was *P* < 0.05.

## Results

We selected 43 patients to include in each of the the intervention and control groups, but we excluded ten patients from each group during the study and analyzed 33 patients. The study results indicated that the mean ages of the participants in the intervention and control groups were 25.39 ± 4.68 and 26.45 ± 4.82, respectively. We found no significant difference in age between the intervention and control groups. Table 2 presents demographic variables and information related to the disease in the intervention and control groups.

**Table 2:** Distribution of absolute and relative frequency (percentage) of qualitative background variables in patients with thalassemia

Group	Intervention		Control		Test	*p*-value
**Variable**	**Frequency**	**Percent**	**Frequency**	**Percent**		
**Marital status**						
Single	26	78.8	22	71.0	4.74^a^	0.07
Married	4	12.1	9	29.0		
Divorced	3	9.1	0	0		
**Children No**						
0	29	87.9	29	87.9	-	-
1–3	4	12.1	4	12.1		
**Age of the first transfusion**						
2 months-one year	24	72.7	25	75.8	0.80^b^	0.78
Above one year	9	27.3	8	24.2		
**History of hospital stay in the last year (day)**						
Yes	17	51.5	14	42.4		
No	16	48.5	19	57.6	0.55^b^	0.46
**Education level**						
Middle/high school	9	27.3	10	30.3	1.48^b^	0.67
Diploma	17	51.5	16	48.5		
Associate degree	3	9.1	1	3.0		
Bachelor’s/higher	4	12.1	6	18.2		
**Sex**						
Female	22	66.7	20	60.6	0.26^b^	0.61
Male	11	33.3	13	39.4		
**Job**						
Unemployed	9	30.0	9	28.1	1.80^b^	0.61
Employed	2	6.7	3	9.4		
Housewife	4	13.3	8	25.0		
Self-employed	15	50.0	12	37.5		
**History of chronic diseases**						
Yes	1	3.0	5	15.2	2.93^a^	0.2
No	32	97.0	28	84.8		

^a^Fisher’s exact test

^b^Chi-square test

The mean rejection sensitivity scores of the intervention group were 8.75 ± 4.86 and 7.11 ± 4.13 before and after the intervention, respectively. The mean rejection sensitivity scores in the control group were 9.87 ± 5.16 and was 10.23 ± 4.94 before and after the intervention, respectively. The homogeneity of regression slopes, which means that there is no interaction between the covariate and the independent variable was confirmed (F = 0.83, *p* = 0.36). The marginal means of rejection sensitivity scores after the intervention in the intervention and control groups were 7.40 and 9.94 respectively. The results of analysis of covariance confirmed that by controlling the effect of pretest score, there was a significant difference between the two groups after the intervention (F = 7.52, *p* = 0.008, Effect size = 0.11). According to the effect size, 11% of the variance of rejection sensitivity score after the intervention is explained with independent variable i.e., group, which is small (Table 3).

**Table 3:** The results of Analysis of Covariance (ANCOVA) for the rejection sensitivity score after the intervention

Source	Type III Sum of Squares	df	F	*P* value	Effect size
Corrected Model	603.32	2	21.50	< 0.001	0.41
Intercept	207.97	1	14.82	< 0.001	0.19
Pretest score	441.87	1	31.49	< 0.001	0.33
Group	105.52	1	7.52	0.008	0.11

R Squared = 0.41 (Adjusted R Squared = 0.39)

The mean resilience scores of the intervention group were 67.72 ± 17.98 and 74.18 ± 17.46 before and after the intervention respectively. The mean resilience scores of the control group were 63.69 ± 19.43 and 58.06 ± 22.81 before and after the intervention, respectively. The homogeneity of regression slopes showed there was no interaction between the covariate and the independent variable (F = 0.35, *p* = 0.56). The marginal means of resilience scores after the intervention in the intervention and control groups were 72.85 and 59.51 respectively. The results of analysis of covariance confirmed that by controlling the effect of pretest score, there was significant difference between two groups after the intervention (F = 9.28, *p* = 0.004, Effect size = 0.15). According to the effect size, 15% of the variance of resilience score after the intervention is explained with independent variable i.e., group, which is small (Table 4).

**Table 4:** The results of Analysis of Covariance (ANCOVA) for the resilience score after the intervention

Source	Type III Sum of Squares	df	F	*P* value	Effect size
Corrected Model	12,445.04	2	24.45	< 0.001	0.49
Intercept	1637.72	1	6.44	0.01	0.11
Pretest score	8941.91	1	35.14	< 0.001	0.41
Group	2360.61	1	9.28	0.004	0.15

R Squared = 0.49 (Adjusted R Squared = 0.47)

## Discussion

The present study aimed to determine the impact of mindfulness-based stress reduction counseling on rejection sensitivity and resilience of the patients with thalassemia and showed a reduction in the mean score of rejection sensitivity compared to before the intervention, and it was statistically significant. Joss et al. (2020) [21] in Nashville supported our results and demonstrated that mindfulness-based counseling was effective in reducing rejection sensitivity, posttraumatic stress disorder, increasing non-attachment and mindfulness in adults with a history of childhood maltreatment [21]. Thalassemia is a genetic chronic disease that includes psychological problems, such as low self-esteem, high anxiety and depression, and physical problems from birth. Patients with thalassemia are pale and have physical problems related to their appearance and bone shape, so they avoid social gatherings and show sensitivity to those around them [3, 4, 12]. According to the study by Joss et al. (2020) [21]—nonattachment predicts empathy, rejection sensitivity and symptom reduction after a mindfulness-based intervention among young adults with a childhood maltreatment history—rejection sensitivity is manifested from the moment of birth and thalassemia also accompanies the patient through the end of life and his/her rejection sensitivity is likely to increase [21]. In mindfulness, people are taught to accept experiences as they are, rather than denying and rejecting unpleasant experiences, and to be aware of themselves and their reactions to unpleasant experiences. In MBSR intervention, they are trained to focus on and improve themselves and their relationships with others in the group form. These exercises will lead to a decrease in rejection sensitivity [36]. Also, mindfulness components such as non-Jugging, acceptance and self-compassion can lead to non-attachment, which itself plays a role in reducing rejection sensitivity [21].

The study results indicated that mindfulness-based stress reduction counseling was effective in increasing the resilience of patients with thalassemia. Resilience is the ability to have adaptive responses to adversities, such as chronic illness, and situations known to be the generators of stress associated with the condition. Resilience can be seen in all kinds of behaviors, thoughts and actions throughout the life cycle [37]. Jabbarifard et al. (2019) were the only ones who measured the impact of mindfulness-based cognitive therapy counseling on the resilience of patients with thalassemia and reported that mindfulness-based cognitive therapy reduced perceived stress, improved resilience and quality of life in patients with thalassemia [15]. Other researchers measured the impact of mindfulness-based stress reduction counseling on resilience in patients with cancer [24], systemic lupus erythematosus [26], vulnerable women [18], and employees [38]. Seyf Hosseini et al. (2019) found that mindfulness-based stress reduction counseling was effective in increasing the resilience of mothers of children with cancer [39]. Naseri Garagoun et al. (2021) showed that mindfulness-based stress reduction counseling increased significantly resilience and life expectancy in patients with gastrointestinal cancers [24]. Bahreini et al. (2019) indicated that mindfulness-based stress reduction counseling increased the resilience of patients with systemic lupus erythematosus and reduced their self-discrepancy [26]. Adelian et al. (2021) revealed that mindfulness-based stress reduction counseling was effective in increasing the resilience of vulnerable women [18]. Diachenko et al. (2021) [38] supported our results and indicated that mindfulness-based stress reduction counseling increased resilience, well-being and life satisfaction of 82 healthy employees aged 50–60 years within 12 months after the intervention. This study was the only one in the literature review in which three out of eight MBSR counseling sessions were held online due to the spread of COVID-19 [38]. We suggest using mindfulness-based stress reduction counseling for thalassemia. Kabat-Zinn [40] also suggested the effect of mindfulness-based interventions, so further studies are necessary to prove its effectiveness and develop it in the future [9]. Improving resilience helps people to acquire better thinking, self-management skills and more knowledge. Resilience with supportive relationships of parents, peers and others, as well as cultural and traditional beliefs, helps people to deal with the inevitable traumas of life [36, 37].

## Limitations

The sampling was limited to the Kerman thalassemia center, so we should be cautious when generalizing the results to people outside the research community. Participants were unwilling to participate in the study because the counselling was online, and the researcher had no physical presence. Some patients had unfavorable physical and mental condition due to the nature of thalassemia, so they were reluctant to continue the study. Due to the conditions of COVID-19 and the reasons mentioned in the previous sentences, it was not possible to follow up with the patients. As the patients in the intervention and the control groups went to the same thalassemia center for blood sampling, they might have exchanged the counseling information, which was another limitation of the present study. In addition, as the effect sizes were small, the interpretation of the results should be with cautions and other studies with larger samples size should conduct to confirm the efficacy of the intervention.

## Conclusion

The study results showed that mindfulness-based stress reduction counseling had a reducing effect on rejection sensitivity of the patients with thalassemia and increased their resilience. We recommend counselors, nurses and managers of thalassemia center and other hospital units to benefit from mindfulness-based stress reduction counseling to reduce patients’ rejection sensitivity and improve patients’ resilience. We suggest studies be conducted on the effect of mindfulness-based stress reduction counseling on other psychological problems of patients with thalassemia, such as self-esteem, depression, social isolation, loneliness level in different cultures and contexts.

## Data Availability

The datasets supporting the conclusions of this article are available from the corresponding author on reasonable request.
